# Chemical Composition and Nutritive Value of Almond Hulls from Two Almond Varieties and Influence of Including Almond Hulls in the Diet on In Vitro Ruminal Fermentation and Methane Production

**DOI:** 10.3390/vetsci11060242

**Published:** 2024-05-29

**Authors:** Adriana Recalde, Trinidad de Evan, Carlos Fernández, Rafael A. Roldán, Silvia López-Feria, María Dolores Carro

**Affiliations:** 1 Departamento de Producción Agraria, ETSIAAB, Universidad Politécnica de Madrid, Ciudad Universitaria, 28040 Madrid, Spain; adriana.recalde@alumnos.upm.es (A.R.); t.deevan@upm.es (T.d.E.); 2 Instituto de Ciencia y Tecnología Animal, Universitat Politècnica de València, 46022 Valencia, Spain; cjfernandez@dca.upv.es; 3 Grupo de Prado. Avda. Cervantes 6, 14008 Córdoba, Spain; rafaelroldan@deprado.eu (R.A.R.); silvia.lopez@dcoop.es (S.L.-F.); 4 Departamento I + D + i Dcoop S.C.A., Carretera Córdoba s/n, 29200 Antequera, Spain

**Keywords:** almond hulls, energy, ruminal fermentation, in vitro, gas production, methane

## Abstract

**Simple Summary:**

Almond hull (AH) is a by-product of almond production that can be used in ruminant feeding, but their composition is variable and needs further characterization. We observed that there were significant differences in chemical composition and nutritional value of samples of AH from two varieties, with AH from Guara variety having more energy and protein than those from Soleta variety in samples collected in two different almond campaigns in the South of Spain. Moreover, harvesting year affected the chemical composition and nutritive value of AH, making necessary their chemical analysis before being used as feed in the practice. In addition, we conducted in vitro trials to investigate the effects of including increasing amounts of AH in diets for dairy ruminants on diet degradability and methane production. Methane is a greenhouse gas with high warming potential that is produced during feed fermentation in the rumen. The results indicate that dried AH can replace up to 16% of conventional feed ingredients in diets for dairy ruminants, but methane production was not reduced. In conclusion, the tested AH can be used as alternative feed for ruminants, although this by-product does not contain antimethanogenic compounds.

**Abstract:**

Almond hulls (AH) are frequently used in dairy ruminant feeding, but information on variability of their nutritive value and their potential effects on CH_4_ production is still scarce. The influence of almond variety (Guara vs. Soleta) on chemical composition and energy value of AH was investigated using 10 samples per variety collected in 2 consecutive years. Guara-AH had greater (*p* ≤ 0.015) ash, protein, and fat content, but lower (*p* ≤ 0.001) fiber than Soleta-AH. The metabolizable energy content estimated from chemical composition and in vitro gas production was 8.5% greater for Guara than for Soleta samples. Harvesting year significantly affected most of the chemical fractions. The in vitro ruminal fermentation of diets for dairy ruminants including increasing amounts of dried AH (8, 16 and 24% of the total diet; fresh matter basis) indicated that AH can be included up to 16% of the diet, partially substituting corn, wheat bran and sugar beet pulp without detrimental effects on in vitro volatile fatty acid (VFA) production. In contrast, when AH replaced alfalfa hay and corn, VFA production was reduced at all levels of AH inclusion. No antimethanogenic effects of AH were detected in the in vitro incubations.

## 1. Introduction

The demand for almonds (*Prunus dulcis*) is steadily increasing due to the reported health benefits of this fruit and the growing population using almond milk as an alternative to dairy milk. Almond cultivation and processing for human consumption generate several by-products, almond hulls (AH) being the most abundant. The AH is the outer covering of the nut and the hard shell, which can represent about 50% of the weight of the total fruit on a fresh matter basis [1,2], although this value can range from 41.5 to 70.0% for different almond varieties [3]. Almond hulls are rich in carbohydrates, including high proportions of sugars, and are widely used as feed for dairy cows, although several studies have also evaluated their use in small ruminant feeding [4,5]. Chemical composition and nutritive value of AH are influenced by several factors such as cultivation conditions, growing region, harvesting methods, hulling procedures, and almond variety, among others [6]. Although some studies have compared different varieties of AH in the USA [6,7] and Iran [1], samples were collected from different growing regions, and this may have influenced the comparison.

Several studies have investigated the effects of replacing conventional feeds with AH in the diet of dairy cows [8], dairy goats [4], and lambs [5] on animal performance and quality of both milk and meat. However, there is limited information on the influence of AH on ruminal fermentation, and results on its potential effect on CH_4_ production are controversial. Durmic et al. [9] observed that AH could reduce in vitro CH_4_ production without negatively affecting volatile fatty acids (VFA) production, which was attributed to the presence of bioactive compounds such as phenolics and triterpenoids in AH. In contrast, Williams et al. [9] observed that replacing alfalfa cubes with AH in the diet of dairy cows did not affect CH_4_ emissions.

The first objective of this study was to compare the chemical composition and nutritive value of samples of two varieties of AH (Guara and Soleta) collected from a single region and from cultivars grown under the same conditions. The second objective of this study was to assess the influence of including increasing levels of AH in dairy sheep diets on in vitro ruminal fermentation and CH_4_ production. It was hypothesized that AH could replace conventional feed ingredients without negatively affecting ruminal fermentation and might reduce CH_4_ generation.

## 2. Materials and Methods

All the experimental procedures using animals in this study were approved by the Institutional Animal Care and Use Committee of the Comunidad Autónoma de Madrid (Approval number PROEX 132.6/21). Animal care and ruminal sampling followed the European regulations for experimental animal protection.

### 2.1. Almond Hull Samples and In Vitro Incubations

Samples of AH were obtained at almond orchards located at Écija in the South of Spain (O5°4′57.36″ N37°32′31.92″). Five contiguous plots from each almond variety (Guara and Soleta) were harvested in both the 2020 and 2021 campaigns using the most common harvesting procedure in Spain, making a total of 10 samples of Guara and 10 of Soleta AH (5 of each almond variety per harvesting year). Briefly, almond fruits were dropped off the tree using shaking machines and left on the floor until almonds reached the desired humidity level. Then, the fruits were swept into a windrow and harvested. About 5 kg of AH were sampled, frozen (−20 °C), and sent to the laboratory for analysis. Samples were cleaned to eliminate debris (almond shells, sticks, leaves, etc.) and dried at low temperature (40 °C; 72 h) before conducting the analyses of chemical composition and in vitro incubations.

Samples of AH were ground to pass a 1 mm screen and incubated in vitro with ruminal fluid to measure both gas production kinetics and ruminal parameters. Four rumen-fistulated Lacaune sheep (65.0 ± 1.32 kg body weight) were used as donors of ruminal content. Sheep were housed in floor pens with straw bedding and free access to fresh water. A diet composed of 80% grass hay and 20% concentrate (fresh matter basis) was fed twice daily at a daily rate of 45 g dry matter (DM)/kg body weight^0.75^ to avoid diet selection. About 500 g of rumen content was sampled from each sheep via the rumen cannula immediately before the morning feeding and strained through 4 layers of cheesecloth. The fluid was transported to the laboratory into thermal flasks within 20 min after collection, where it was mixed with the buffer solution of Goering and Van Soest [10] in a 1:4 ratio, and the mixture was maintained at 39 °C and flushed with CO_2_.

In vitro incubations were performed as described by De Evan et al. [11]. Briefly, samples of AH and experimental diets (500 mg of DM) were accurately weighed into glass vials of 120 mL volume. Fifty mL of the ruminal fluid and culture medium mixture were added to each vial using a peristaltic pump (520UIP31 model; Watson-Marlow Fluid Technology Group, Barcelona, Spain), and the vials were capped with rubber stops and incubated at 39 °C. In all in vitro runs, the ruminal fluid from each sheep was independently mixed with the culture medium to obtain 4 replicates for each sample, and one vial per sample was incubated.

The incubations for measuring gas production kinetics lasted for 120 h, and the amount of gas produced was measured at 2, 4, 6, 9, 12, 24, 30, 36, 48, 60, 72, 96, and 120 h using a pressure transducer (Delta Ohm DTP704-2BGI; Herter Instruments SL, Barcelona, Spain) and a plastic syringe. The gas produced at each sampling time was released after measurement. Two in vitro runs were conducted, one for the AH from each harvesting year. In each run, 2 blanks (vials without substrate) were incubated for each mixture of ruminal fluid and culture medium (8 blanks in total) to correct gas production values for the gas generated in the fermentation of the substrates added with the ruminal fluid from each sheep.

The incubations for measuring ruminal parameters lasted for 24 h. In order to detect possible differences among samples at short incubation times, after 6 h of incubation, a sample (1 mL) of each vial content was taken using an insulin syringe, which was mixed with 1 mL of 0.5 M HCl and immediately frozen (−20 °C) until analysis of volatile fatty acid (VFA) and NH_3_-N concentrations. After 24 h of incubation, the gas production was measured, vials were hand-shaken, uncapped, the pH of their content was measured (Crison Basic 20 pH meter; Crison Instruments, Barcelona, Spain), and 1 mL of content was taken and processed as previously described for analysis of VFA and NH_3_-N concentrations. Two in vitro runs were conducted, one for the AH samples collected at each harvesting year.

### 2.2. Experimental Diets and In Vitro Incubations

The objective of these incubations was to assess the effects of including increasing amounts of dried AH in diets for dairy sheep on in vitro rumen fermentation and CH_4_ production. Two diets for dairy sheep were formulated without AH (control diets): one for high-producing sheep (HP0) and the other for medium-producing sheep (MP0). For each type of diet, 3 additional diets were formulated by replacing different amounts of feed ingredients with 8, 16, or 24% (fresh matter basis) of dried AH. The AH sample was from Soleta variety and contained 92.3, 6.83, 36.7, 26.6, 4.58, and 23.8 g of organic matter (OM), crude protein (CP), neutral detergent fiber (NDF), acid detergent fiber (ADF), ether extract (EE), and total sugars per 100 g of DM, respectively.

Diets were formulated to meet the nutritive requirements of high- and medium-production dairy sheep according to INRA Feeding System [12]. Within HP and MP diets, all diets were formulated to have similar CP and NDF content. Feed ingredients and chemical composition of the experimental diets formulated for high-producing sheep (HP diets) are shown in Table 1. In these diets, the AH partly replaced corn, wheat bran, and sugar beet pulp.

As shown in Table 2, in the diets formulated for medium-producing dairy sheep (MP diets), the AH partly replaced alfalfa hay and corn, and urea was included in the diets containing AH to achieve isonitrogenous diets.

The in vitro incubations of experimental diets were conducted following the same methodology described for AH samples, with the exception that the composition of the culture medium of Goering and Van Soest [10] was modified to exclude all N-containing chemicals, as the CP content of the diets was adequate to allow optimal microbial growth. Gas production was measured at 9 and 24 h, and a 15 mL sample of the gas was collected in vacuum tubes and stored at room temperature until CH_4_ analysis. Sampling at 9 h of incubation was chosen because previous studies from our group [13,14] showed that in vitro CH_4_ production is very low at shorter incubation times. Samples of all experimental diets were incubated in one run using one vial per sample.

In addition, the potential DM degradability (PDMD) of each diet was determined by weighing 300 mg of each diet into Ankom Corp #57 polyester bags (30 µm pore size; Ankom Technology Corp., Fairport, NY, USA) in duplicate. Bags were heat-sealed and incubated in an Ankom Daisy II incubator (Ankom Technology Corp., Fairport, NY, USA) at 39 °C in the 1:4 mixture of ruminal fluid and the incubation medium of Goering and Van Soest [10] previously described under continuous rotation. After 120 h, the bags were washed with cold water, dried at 60 °C for 48 h, and weighed to calculate the PDMD. This value was used to estimate the effective DM degradability (DMED) as described below.

### 2.3. Chemical Analyses

Chemical composition of AH samples and the feed ingredients of the experimental diets were analyzed as described by Marcos et al. [15]. Briefly, the content in DM, ash, crude fiber (CF), and EE was analyzed using the Association of Official Analytical Chemists (AOAC) [16] procedures, whereas NDF, ADF, and lignin content was determined according to Van Soest et al. [17]. The Dumas combustion method was used to measure N content, and total sugar content was determined by spectrophotometry according to the Folin–Ciocalteu assay [18]. The concentration of Ca, Mg, K, Na, and Cu in the AH samples was determined by atomic absorption spectrometry according to the ISO (International Organization for Standardization) 6869 using a Varian SpectrAA 220FS atomic absorption spectrophotometer (Varian Medical System Ibérica, Madrid, Spain) [19]. Phosphorus concentration was determined by a colorimetric method [20] using an ESPECORD^®^ 200 plus (Analytik Jena GmbH, Jena, Germany) spectrophotometer.

Concentrations of NH_3_-N in vials content were determined by the phenol-hypochlorite method [21], and those of VFA and CH_4_ were analyzed by gas chromatography using a flame ionization detector (FID) as described by Tejido et al. [22] and Martínez et al. [23], respectively. An Epoch spectrophotometer (BioTek Instruments Inc., Winooski, VT, USA) was used for total sugar and NH_3_-N determinations. All chemical analyses were done in duplicate.

### 2.4. Calculations and Statistical Analyses

Data on gas production for each sample (AH and experimental diets) were fitted with time according to the model described by Krishnamoorthy et al. [24]: Gas = PGP × (1 − e^(−*c*×(t−*Lag*))^). In this model, PGP is the potential gas production, *c* is the fractional rate of gas production, *Lag* is the time before starting gas production, and t is the time of gas measurement. Data fitting to the model was achieved with the NLIN PROC of the statistical package SAS [25] using an iterative least squares procedure. In addition, the average gas production rate (AGPR) was calculated using the following equation proposed by France et al. [26]: AGPR = PGP × c/[2 × (ln2 + *c* × *Lag*)]. The AGPR is the rate of gas production between the incubation start and the time at which gas production reaches 50% of PGP. The DMED was estimated for a rumen particulate outflow (Kp) of 6% per h from PDMD and gas production parameters as: DMED = [(PDMD × *c*)/(*c* + Kp)] e^(−*c*×*Lag*)^.

The metabolizable energy (ME) content of AH samples was estimated from CP and EE content (expressed as g/kg DM) and the gas produced after 24 h of incubation from 300 mg of DM incubated (G24) using the following equation proposed by Menke and Steingass [27]: ME = 2.43 + 0.1206 × G24 + 0.0069 × CP + 0.0187 × EE. Finally, the amount of apparently fermented OM (AFOM) for each experimental diet was estimated from the amount of acetate, propionate, and butyrate produced in each vial as proposed by Demeyer [28].

All statistical analyses were conducted using the SAS package [25]. Data on chemical composition and energy value of AH were analyzed as a mixed model with repeated measures over time using the PROC MIXED of SAS. The model included the almond variety, harvesting year, and their interaction as fixed effects, and the cultivation plot as random effect. Gas production and ruminal parameters were analyzed using the same model, but additionally including the rumen inoculum (mixture of rumen fluid and culture medium) as random effect.

Data on gas production and fermentation parameters of experimental diets were analyzed independently for each type of diet (HP or MP) and sampling time (9 and 24 h of incubation). Data were analyzed as a mixed model, with the fixed effect of the diet and the random effect of the rumen inoculum. Non-orthogonal polynomial contrasts were used to test for linear and quadratic effects of the inclusion of AH. When a significant effect of AH inclusion was detected, means were compared by Tukey’s test. For all statistical analyses, significance was established at values of *p* < 0.05, and values of *p* < 0.10 were treated as trends.

Finally, relationships between chemical fractions of AH samples and either gas production or fermentation parameters were assessed by linear regression using the PROC CORR of SAS [25].

## 3. Results and Discussion

### 3.1. Influence of Almond Variety on Nutritive Value of Almond Hulls

Table 3 shows the chemical composition and energy content of AH from Soleta and Guara varieties harvested in two different years. There were significant differences (*p* < 0.05) between almond varieties in all chemical fractions analyzed, excepting EE and total sugars content. Almond hulls from Guara variety had greater (*p* ≤ 0.015) DM, ash, CP, and CF content, but lower (*p* ≤ 0.001) NDF, ADF, and lignin content and lignin/NDF ratio than AH from Soleta variety. As a consequence, the estimated ME content and UFL and UFV values were greater (*p* ≤ 0.003) for Guara compared with Soleta AH. Previous studies have also reported significant differences in the chemical composition of AH due to almond variety both in California (Merced, Nonpareil, and Neplus varieties) [7] and in Iran (Rabbi, Mamaii, Shahrud, and Shokufe varieties) [29]. Our data indicate that differences in chemical composition were maintained in the two harvesting years, as there were no variety x harvesting year interactions (*p* ≥ 0.05) for any chemical fraction except for EE content (*p* < 0.001).

The chemical composition of AH is very variable and is affected by multiple factors, such as the variety of almond, fertilizer treatment, cultivation conditions, maturity, weather conditions at harvesting, and procedure of almond harvesting, among others [6]. Harvesting year affected all chemical fractions excepting EE and total sugars content and lignin/NDF ratio. For both almond varieties, samples collected in 2020 contained more DM, ash, and CP, and less NDF, ADF, and lignin than those harvested in 2021. In contrast, EE of Guara-AH was greater in 2021 than in 2020 samples, but for Soleta-AH, it was greater in 2020 compared with 2021 samples. In general, chemical composition of both AH varieties was in the range of values reported by Feedipedia [30] and in the Tables of INRA [12] and NRC [31], except for CP content that was slightly higher in our study, especially for Guara samples.

The greater (*p* < 0.001) ME content of Guara-AH compared with Soleta-AH is consistent with the lower content in fiber (NDF, ADF, and CF fractions) and lignin and the greater CP content of Guara (Table 3). In fact, the ME level of the 20 AH samples was negatively correlated with their content in NDF (r = −0.839; *p* < 0.001), ADF (r = −0.982; *p* < 0.001), CF (r = −0.515; *p* = 0.020), and lignin (r = −0.776; *p* < 0.001), and positively correlated with CP content (r = 0.666; *p* = 0.001). For both AH varieties, samples harvested in 2021 had lower ME content (*p* < 0.001) than those collected in 2020, which agrees well with the greater fiber and lignin content of the samples harvested in 2021.

The average ME content of all samples (2096 kcal/kg DM) is greater than that reported by NRC [31], but agrees well with the value of 2078 kcal ME/kg DM reported by Feedipedia [30]. Similarly, the average values of UFL and UFV for the 20 AH samples analyzed in the present study (0.74 and 0.66 per kg DM, respectively; Table 3) are comparable to the 0.75 UFL and 0.66 UFV values reported by INRA [12]. These results indicate that AH collected in Spain have a similar energy value for ruminants as those produced in other Mediterranean countries, and that AH are a medium-quality energy feed for ruminants.

The two AH varieties analyzed in the present study showed some differences in mineral content, with AH from Guara variety having more (*p* < 0.001) Ca, Mg, and K, but less P (*p* = 0.010) than Soleta-AH (Table 3). In contrast, no differences (*p* ≥ 0.300) between varieties were detected in Na and Cu content. The content in minerals was similar to that reported previously by Feedipedia [30], NRC Tables [31], and other studies analyzing different AH varieties [1,6,29], confirming the high K content of AH compared to forages and fibrous by-products such as cereal bran [12,30,31]. Harvesting year affected (*p* ≤ 0.025) the content of all minerals analyzed, excepting Mg and Cu, despite that the same fertilizer treatment being applied to each plot in both years. Table 4 shows the gas production and fermentation parameters of the AH samples after in vitro incubation with ruminal fluid from sheep. The lower (*p* < 0.001) PGP and AGPR values of Soleta compared with Guara AH can be explained by the greater NDF and ADF content of Soleta, as fiber is less fermentable in the rumen than other chemical fractions (i.e., sugars, soluble carbohydrates, CP), and therefore generates less gas. However, in the analysis of relationships among gas production parameters and chemical composition (Table 5), no significant correlations (*p* ≥ 0.266) between PGP and either fiber (NDF, ADF, and CF fractions) or CP content were detected. In contrast, PGP was negatively correlated with lignin content (r = −0.489; *p* = 0.029) and lignin/NDF ratio (r = −0.607; *p* = 0.005), indicating that lignin limited the ruminal degradability of AH, as generally observed for ruminant fibrous feeds [32].

Almond hulls from Soleta variety presented lower (*p* < 0.001) *Lag* times than Guara-AH over both harvesting years, indicating that rumen degradation started more rapidly for Soleta samples. *Lag* time is usually negatively related to the content of rapidly degradable substances such as sugars and easily fermented carbohydrates [33]. However, in the present study, no correlation was detected between *Lag* time and sugar content (r = 0.185; *p* = 0.426), suggesting that other chemical fractions, not analyzed in the present study, were responsible for the reduced *Lag* time observed for Soleta AH. *Lag* was positively correlated with CP content and negatively with the content in NDF, ADF, and lignin, which are fractions more slowly degraded (NDF and ADF) or undegradable (lignin). Similarly, the AGPR was negatively correlated with NDF, ADF, and lignin content and the lignin/NDF ratio (Table 5).

In agreement with the observed differences in PGP, the fermentation of Guara samples generated more total VFA than Soleta samples at both 6 and 24 h of in vitro incubation. The negative relationships observed between VFA production at 9 and 24 h and the lignin/NDF ratio (Table 5) are consistent with the negative effect of lignin on fiber degradation in the rumen [32]. As observed previously for other feeds [33], PGP was positively correlated with total VFA production at 6 and 24 h of incubation (r = 0.762 and 0.774, respectively; *p* < 0.001). The lower pH values (*p* < 0.001) observed for Guara than for Soleta also indicate greater fermentability of Guara samples, and pH values were negatively correlated with total VFA production (r = −0.942; *p* < 0.001; n = 20) when both were measured after 24 h of incubation.

The VFA profile differed between almond varieties, with Guara AH resulting in greater (*p* < 0.001) molar proportions of acetate and lower (*p* ≤ 0.007) of propionate, butyrate, and isovalerate than Soleta AH at 6 h of incubation. Differences between AH varieties were reduced at 24 h of incubation, but Guara AH still produced greater (*p* < 0.001) proportions of acetate and lower (*p* < 0.001) of butyrate and isovalerate compared with Soleta. The greater propionate proportions of Soleta at 6 h of incubation could be due to the presence of soluble and rapidly degraded fractions that contribute to propionate production, and this hypothesis is consistent with the lower *Lag* time of Soleta AH. At 6 h of incubation, Guara samples showed greater (*p* < 0.001) acetate/propionate ratios than Soleta, but differences disappeared after 24 h of incubation (*p* = 0.527). We are not aware of studies reporting the fermentation profile of AH, but Durmic et al. [9] observed that adding AH to a control mixed diet (1:1 ratio) resulted in a significative increase in acetate/propionate ratio after 24 h of in vitro incubation, which agrees with the high acetate proportions observed in the present study for all AH samples. Similarly, Rad et al. [5] observed no changes in acetate and propionate proportions and acetate/propionate ratio in the rumen of lambs when AH was included up to 40.0% in the diet replacing a fibrous feed (alfalfa hay).

Despite of the greater CP content in Guara than in Soleta samples, there were no differences between AH varieties in NH_3_-N concentrations (*p* ≥ 0.324) at any incubation time. However, there was a positive correlation (r = 0.830; *p* < 0.001; Table 5) between NH_3_-N concentrations at 24 h and CP content of AH samples. Ruminal CP degradability of AH has been reported to be low due to the high proportion of CP linked to the ADF [12,31]. In the present study, NH_3_-N concentrations were adequate for in vitro microbial growth [34] due to the use of a N-enriched culture medium for the in vitro incubations. Moreover, it has to be noted that the measured NH_3_-N concentrations are the balance between the NH_3_-N generated in the degradation of CP and the NH_3_-N captured by the microbiota for microbial protein synthesis.

### 3.2. Effects of Including Almond Hulls on In Vitro Ruminal Fermentation of Dairy Sheep Diets

Several studies have investigated the effects of replacing conventional feed ingredients in ruminant diets with AH, but results have been inconsistent [4,5,8,9]. Controversial results from different studies can be attributed to variations in the nutritive value of AH and their inclusion level in the diet, but also to differences in the quality of feeds being replaced in the diets. Therefore, the second part of this study was designed to assess the effects of including increasing amounts of AH replacing different feeds in the diet. Almond hulls partly replaced concentrate ingredients (corn, wheat bran, and sugar beet pulp) in HP diets, whereas in MP diets, AH mostly replaced forage (alfalfa hay; 60.0% in MP0 to 40.3% in MP24 diet) and only a little proportion of corn (15% in MP0 to 10.2% in MP24 diet). Within HP and MP, all experimental diets were formulated to contain similar N and NDF levels. In addition, the culture medium of Goering and Van Soest [10] used in the in vitro incubations was modified to exclude the N-containing compound (NH_4_HCO_3_) to avoid extra N supplementation.

As shown in Table 6, including AH in HP diets did not affect any production parameter except for the fractional gas production rate (*c*) that was linearly reduced (*p* < 0.001) as dietary AH levels increased. Similarly, the DMED was linearly decreased (*p* < 0.001) by increasing the amount of AH in the diet. At 9 h of incubation, VFA production was unaffected *(p* > 0.05) by AH inclusion, but the VFA profile was modified. Propionate proportions were linearly increased (*p* = 0.027), and valerate proportions (*p* = 0.003) and acetate/propionate ratios (*p* = 0.028) were linearly reduced by increasing AH levels in the diet, although only the HP24 diet showed significant differences (*p* < 0.05) with the control diet. The increase in propionate proportions observed in the diets with AH is consistent with the presence of sugars and other rapidly degradable substances in AH. Neither the amount of AFOM nor CH_4_ production and CH_4_/total VFA ratio were influenced (*p* ≥ 0.303) by including AH in the diet, indicating that diet fermentation was relatively unchanged at 9 h of incubation.

In contrast, after 24 h of incubation, more differences among diets were detected. Both total VFA production and the amount of AFOM were linearly reduced (*p* = 0.008, and 0.004, respectively) and pH was linearly increased (*p* < 0.001) by dietary AH inclusion, indicating a reduction of diet fermentability when AH were included. In addition, butyrate proportion linearly decreased (*p* = 0.042) and that of propionate tended to rise (*p* = 0.087) with increasing AH levels. Altogether, these results might indicate that some AH fractions were more rapidly fermented than those in some of the replaced feeds (corn, wheat bran, and sugar beet pulp), resulting in no differences in VFA production or AFOM at 9 h of incubation, but afterwards, AH were less fermented, resulting in reduced VFA production for diets containing AH.

There were no differences among diets in NH_3_-N concentrations at 9 h of incubation, but after 24 h, NH_3_-N concentrations were linearly reduced (*p* = 0.026; Table 6) by including AH in the diet. The observed reduction in NH_3_-N concentrations is consistent with the reported low degradability of CP of AH [12,31].

The potential effects of AH on ruminal CH_4_ production have been investigated in a limited number of studies, and the results are contradictory. Whereas Durmic et al. [9] observed a reduction in in vitro CH_4_ production when AH were added to a mixed substrate, Williams et al. [9] observed no change in ruminal CH_4_ emissions of dairy cows when dietary alfalfa cubes were partly replaced by AH. In the present study, CH_4_ production was unaffected at 9 h of incubation, but at 24 h of incubation, it was linearly decreased (*p* = 0.013) by increasing the amounts of AH in the diet (Table 6). Although these results might indicate an antimethanogenic effect of AH, the similar CH_4_/total VFA ratios observed for all diets (*p* = 0.642 and 0.376 for linear and quadratic effects, respectively) indicate that the observed reduction in CH_4_ production was due to decreased ruminal fermentability of the diets containing AH. The CH_4_/total VFA ratio can be used as an indicator of ruminal fermentation efficiency [35], as CH_4_ is an energy loss and VFAs are both the main energy source for the host animal and precursors for the synthesis of other compounds such as propionate for glucose synthesis and acetate and butyrate for fatty acids synthesis [32].

Table 7 shows the gas production and fermentation parameters of the diets formulated for medium-producing sheep, in which AH partly replaced alfalfa hay and corn. The inclusion of AH linearly reduced the time at which gas production started (*Lag*; *p* = 0.008) and the fractional rate of gas production (*c*; *p* ≤ 0.001) without affecting PGP and AGPR. The DMED was quadratically decreased (*p* = 0.030) by increasing the amount of AH in the diet.

Similarly to that observed for HP diets, the inclusion of AH in the MP diets did not affect VFA production or the amount of AFOM at 9 h of incubation. However, after 24 h, it resulted in a linear decrease (*p* <0.001) of both total VFA production and AFOM and a linear increase of pH (*p* < 0.001), altogether indicating lower fermentation of AH-containing diets compared with control diet (MP0). Changes in VFA profile were also observed at both measurement times, with VFA production being shifted to propionate at the expense of acetate when AH were included in the diet, with acetate/propionate ratios being lower for the diets with AH. Moreover, proportions of valerate were linearly decreased (*p* < 0.001) at both 9 and 24 h of incubation by using AH as feed ingredient. Decreased proportions of valerate can be related to a reduction in CP degradation or amino acid deamination, as valerate is a breakdown product of proline [36], and these results are consistent with the reduced NH_3_-N concentrations observed at 24 h of incubation when AH were included in the diet.

In accordance with that observed for HP diets, including AH in the diet did not affect either CH_4_ production or CH_4_/total VFA ratios at any measurement time, indicating that the used AH did not contain antimethanogenic compounds.

## 4. Conclusions

Almond variety can affect both chemical composition and nutritional value of almond hulls, and the detected differences between Guara and Soleta varieties were maintained across two harvesting years. The nutritive value of almond hulls was variable between harvesting years, even within the same almond variety. Including almond hulls in ruminant diets can result in variable effects on ruminal fermentation, depending on the inclusion level and the feeds being replaced. In general, the inclusion of AH up to 16% of the diet had little effects on in vitro ruminal fermentation, but greater levels reduced diets fermentability, especially at 24 h of fermentation. No antimethanogenic effects of AH were observed in the present study.

## Figures and Tables

**Table 1:** Ingredients and chemical composition of diets for high-producing dairy sheep without almond hulls (HP0) and with almond hulls at 8 (HP8), 16 (HP16), and 24% (HP24) of fresh matter.

Item	HP0	HP8	HP16	HP24
Ingredients (% fresh matter)				
Alfalfa hay	40.0	40.0	40.0	40.0
Almond hulls	-	8.0	16.0	24.0
Corn	14.0	10.2	6.28	2.41
Wheat	6.00	6.00	6.00	6.00
Barley	10.5	10.5	10.50	10.50
Soybean meal 46.5	7.00	7.00	7.00	7.00
Rapeseed meal 00	5.50	5.50	5.50	5.50
Wheat bran	5.00	3.62	2.24	0.86
Sugar beet pulp	10.0	7.24	4.48	1.72
Calcium soap	1.00	1.00	1.00	1.00
Calcium carbonate	0.50	0.50	0.50	0.50
Mineral/vitamin premix	0.50	0.50	0.50	0.50
Chemical composition ^1^				
Dry matter (DM; %)	91.3	91.3	91.2	91.3
Organic matter (% DM)	92.6	92.3	91.9	91.6
Crude protein (% DM)	16.6	16.6	16.5	16.4
Neutral detergent fiber (% DM)	36.5	37.1	37.7	38.3
Acid detergent fiber (% DM)	21.1	22.3	23.5	24.7
Lignin (% DM)	4.26	5.25	6.18	7.15
Ether extract (% DM)	3.44	3.79	4.13	4.48

^1^ Calculated from chemical analysis of individual feed ingredients.

**Table 2:** Ingredients and chemical composition of diets for medium-producing dairy sheep without almond hulls (MP0) and with almond hulls at 8 (MP8), 16 (MP16), and 24% (MP24) of fresh matter.

Item	MP0	MP8	MP16	MP24
Ingredients (% fresh matter)				
Alfalfa hay	60.0	53.4	46.9	40.3
Almond hulls	-	8.0	16.0	24.0
Urea	-	0.18	0.35	0.55
Corn	15.0	13.4	11.8	10.2
Barley	8.5	8.5	8.5	8.5
Soybean meal 46.5	3.0	3.0	3.0	3.0
Wheat bran	2.5	2.5	2.5	2.5
Sugar beet pulp	9.0	9.0	9.0	9.0
Calcium soap	1.00	1.00	1.00	1.00
Calcium carbonate	0.50	0.50	0.50	0.50
Mineral/vitamin premix	0.50	0.50	0.50	0.50
Chemical composition ^1^				
Dry matter (DM; %)	91.8	91.8	90.9	90.5
Organic matter (% DM)	91.4	91.5	91.5	91.6
Crude protein (% DM)	14.7	14.7	14.7	14.8
Neutral detergent fiber (% DM)	40.9	40.9	40.5	40.1
Acid detergent fiber (% DM)	26.1	26.1	26.0	25.8
Lignin (% DM)	5.18	5.84	6.46	7.11
Ether extract (% DM)	3.22	3.58	3.94	4.30

^1^ Calculated from chemical analysis of individual feed ingredients.

**Table 3:** Influence of harvesting year (HY) and almond variety (VAR) on chemical composition and energy and mineral content of Guara and Soleta almond hulls.

Harvesting Year	2020		2021			*p*=		
Almond Variety	Guara	Soleta	Guara	Soleta	SEM	VAR	HY	VAR*HY
Chemical composition								
Dry matter (DM)	75.4	69.6	67.2	60.6	2.41	0.015	0.007	0.885
Ash	10.9	7.57	9.92	7.15	0.292	<0.001	0.005	0.200
Crude protein	10.1	7.25	7.04	6.45	0.596	<0.001	0.029	0.176
Neutral detergent fiber (NDF)	29.9	34.6	37.9	39.8	0.669	<0.001	<0.001	0.069
Acid detergent fiber	20.9	25.6	27.3	30.0	0.414	<0.001	<0.001	0.071
Lignin	8.40	12.8	10.7	14.4	0.316	<0.001	<0.001	0.277
Lignin/NDF (%)	28.2	36.9	28.4	36.1	0.718	<0.001	0.636	0.323
Crude fiber	15.1	14.5	18.9	15.5	0.652	0.009	0.003	0.097
Total sugars	23.1	23.2	23.1	24.9	1.331	0.527	0.487	0.487
Ether extract	3.26	5.00	5.01	3.80	0.330	0.502	0.332	<0.001
Energy content (kcal/kg DM)								
ME (kcal/kg DM) ^1^	2275	2115	2088	1907	21.3	<0.001	<0.001	0.678
UFL (per kg DM) ^2^	0.82	0.74	0.73	0.65	0.009	<0.001	<0.001	0.921
UFV (per kg DM) ^2^	0.76	0.66	0.65	0.56	0.010	<0.001	<0.001	0.629
Minerals								
Calcium (g/kg DM)	4.44	2.36	5.15	2.34	0.241	<0.001	0.025	0.021
Phosphorus (g/kg DM)	1.58	1.74	1.13	1.44	0.107	0.010	0.015	0.600
Magnesium (g/kg DM)	2.04	1.19	2.11	1.18	0.055	<0.001	0.529	0.378
Potassium (g/kg DM)	45.7	33.0	36.0	27.9	1.58	<0.001	<0.001	0.108
Sodium (g/kg DM)	0.56	0.55	0.53	0.54	0.002	0.539	<0.001	0.054
Copper (mg/kg DM)	5.55	5.51	6.19	5.68	0.258	0.300	0.145	0.394

^1^ Estimated from chemical composition and gas production at 24 h incubation as described by Menke and Steingass [27]; ^2^ UFL (forage units for milk production) and UFV (forage units for meat production) were calculated according to INRA Feeding System [12].

**Table 4:** Influence of almond variety (VAR) and harvesting year (HY) on gas production parameters and fermentation parameters of Guara and Soleta almond hulls after 6 and 24 h of incubation with ruminal fluid from sheep.

Harvesting Year	2020		2021			*p*=		
Almond Variety	Guara	Soleta	Guara	Soleta	SEM ^2^	VAR	HY	VAR*HY
Gas production parameters ^1^								
PGP (mL/g dry matter (DM))	219	198	223	214	3.2	<0.001	0.301	0.051
*Lag* (h)	0.234	0.000	0.108	0.004	0.0337	<0.001	0.074	0.059
*c* (%/h)	5.54	5.13	4.39	4.38	0.020	0.306	<0.001	0.338
AGPR (mL/h)	8.42	7.27	6.98	6.73	0.241	0.005	<0.001	0.067
Fermentation parameters (6 h)								
Total volatile fatty acids (VFA; µmol/g DM)	3.21	2.80	3.54	3.14	0.087	<0.001	<0.001	0.964
Molar proportion of VFA (mol/100 mol)								
Acetate (Ac)	61.5	57.5	61.0	56.4	0.82	<0.001	0.338	0.710
Propionate (Pr)	30.9	32.3	31.2	34.5	0.83	0.007	0.138	0.254
Butyrate	6.38	8.56	6.25	7.27	0.306	<0.001	0.024	0.062
Isobutyrate	0.367	0.458	0.969	1.179	0.1963	0.445	0.001	0.762
Isovalerate	0.128	0.622	0.052	0.111	0.0340	<0.001	<0.001	<0.001
Valerate	0.673	0.589	0.469	0.554	0.0563	0.993	0.038	0.138
Ac/Pr (mol/mol)	2.01	1.80	2.01	1.68	0.075	<0.001	0.413	0.464
NH_3_-N (mg/L)	68.4	55.7	73.3	71.3	7.36	0.324	0.166	0.472
Fermentation parameters (24 h)								
pH	6.81	6.86	6.74	6.75	0.008	<0.001	<0.001	0.025
Total VFA (µmol/g DM)	6.40	5.20	7.47	6.97	0.150	<0.001	0.007	0.022
Molar proportion of VFA (mol/100 mol)								
Acetate (Ac)	62.7	61.1	62.2	59.6	0.45	<0.001	0.033	0.264
Propionate (Pr)	28.7	26.7	26.8	27.7	0.46	0.226	0.383	0.003
Butyrate	6.94	9.62	7.39	8.87	0.304	<0.001	0.624	0.051
Isobutyrate	0.331	0.720	2.565	2.618	0.1551	0.162	<0.001	0.285
Isovalerate	0.536	0.981	0.248	0.394	0.0612	<0.001	<0.001	0.018
Valerate	0.832	0.914	0.716	0.858	0.0875	0.207	0.331	0.733
Ac/Pr (mol/mol)	2.20	2.31	2.33	2.16	0.046	0.527	0.752	0.004
NH_3_-N (mg/L)	124	114	105	109	8.0	0.693	0.155	0.398

^1^ PGP: potential gas production; *c*: fractional rate of gas production; *Lag*: time before starting gas production; AGPR: average gas production rate; ^2^ SEM: standard error of the mean.

**Table 5:** Correlation matrix (Pearson coefficient and *p* values in brackets; n = 20) of the chemical composition of almond hull samples with gas production parameters measured in 120 h in vitro incubations and fermentation parameters after 6 and 24 h of in vitro incubations (only *p* < 0.05 values are shown).

	Gas Production Parameters ^1^				Fermentation Parameters ^2^				
Item	PGP	*Lag*	*c*	AGPR	VFA 6 h	VFA 24 h	NH_3_-N 6	NH_3_-N 24	pH 24
Dry matter		0.579 (0.008)	0.598 (0.005)	0.684 (<0.001)					
Ash		0.761 (<0.001)		0.498 (0.025)	0.508 (0.022)				
Crude protein		0.449 (0.047)	0.536 (0.015)	0.502 (0.024)				0.831 (<0.001)	
Neutral detergent fiber (NDF)		−0.581 (0.007)	−0.804 (<0.001)	−0.854 (<0.001)					−0.449 (0.047)
Acid detergent fiber		−0.670 (0.001)	−0.807 (<0.001)	−0.885 (<0.001)					
Lignin	−0.487 (0.029)	−0.796 (<0.001)	−0.582 (0.007)	−0.812 (<0.001)					
Lignin/NDF	−0.607 (0.005)	−0.736 (<0.001)		−0.529 (0.016)	−0.578 (0.008)	−0.523 (0.018)			
Crude fiber						−0.542 (0.014)			−0.521 (0.018)
Total sugars								−0.640 (0.002)	
Ether extract				−0.456 (0.043)					

^1^ PGP: potential gas production; *c*: fractional rate of gas production; *Lag*: time before starting gas production; AGPR: average gas production rate; ^2^ total volatile fatty acid (VFA) production and NH_3_-N concentrations measured after 6 or 24 h of incubation.

**Table 6:** Gas production and fermentation parameters of diets for high-producing dairy sheep without almond hulls (HP0) and with almond hulls at 8 (HP8), 16 (HP16), and 24% (HP24) of the fresh matter at 9 and 24 h of in vitro incubation with ruminal fluid from sheep.

Item	HP0	HP8	HP16	HP24	SEM ^2^	*p*=	
						Lineal	Quadratic
Gas production parameters ^1^							
PGP (mL/g dry matter (DM))	267	259	256	248	6.1	0.066	0.976
*Lag* (h)	0.060	0.057	0.056	0.056	0.003	0.367	0.621
*c* (%/h)	2.07	1.62	1.28	0.86	0.12	<0.001	0.904
AGPR (mL/h)	9.71	9.34	9.44	9.43	0.42	0.702	0.679
DMED (%)	38.0 ^c^	36.3 ^b^	34.3 ^a^	35.3 ^a^	0.31	<0.001	0.002
Fermentation parameters (9 h)							
Total volatile fatty acids (VFA; mmol/g DM)	3.95	3.94	3.85	3.82	0.087	0.266	0.889
Molar proportion of VFA (mol/100 mol)							
Acetate (Ac)	63.0	62.8	62.2	62.5	0.27	0.141	0.608
Propionate (Pr)	22.2 ^a^	22.2 ^ab^	23.3 ^ab^	23.5 ^b^	0.42	0.027	0.867
Butyrate	10.8	10.6	10.5	10.4	0.15	0.055	0.921
Isobutyrate	0.605	0.697	0.733	0.465	0.0968	0.470	0.170
Isovalerate	2.04	2.35	2.04	1.93	0.046	0.151	0.318
Valerate	1.33 ^b^	1.30 ^ab^	1.21 ^a^	1.22 ^a^	0.021	0.003	0.311
Ac/Pr (mol/mol)	2.85 ^b^	2.84 ^ab^	2.68 ^ab^	2.66 ^a^	0.061	0.028	0.969
NH_3_-N (mg/L)	143	139	141	143	2.5	0.720	0.219
CH_4_ (mL)	18.3	18.6	18.4	18.0	0.39	0.662	0.401
CH_4_/VFA (mL/mol)	4.65	4.75	4.81	4.74	0.111	0.528	0.449
AFOM (mg/g) ^3^	342	328	332	330	7.0	0.303	0.701
Fermentation parameters (24 h)							
pH	6.84 ^a^	6.88 ^b^	6.89 ^bc^	6.91 ^c^	0.007	<0.001	0.171
Total VFA (mmol/g DM)	6.73 ^b^	6.30 ^ab^	6.35 ^ab^	5.98 ^a^	0.146	0.008	0.714
Molar proportion of VFA (mol/100 mol)							
Acetate (Ac)	62.3	62.7	63.0	62.5	0.45	0.699	0.411
Propionate (Pr)	20.1	19.9	20.1	20.8	0.24	0.087	0.116
Butyrate	13.2 ^b^	13.2 ^b^	12.8 ^ab^	12.5 ^a^	0.25	0.042	0.610
Isobutyrate	1.19	1.07	1.06	1.10	0.056	0.327	0.178
Isovalerate	1.75	1.77	1.72	1.80	0.027	0.289	0.627
Valerate	1.35	1.34	1.30	1.32	0.448	0.733	0.642
Ac/Pr (mol/mol)	3.10	3.15	3.13	3.01	0.052	0.264	0.144
NH_3_-N (mg/L)	222 ^b^	222 ^b^	210 ^ab^	206 ^a^	4.8	0.026	0.707
CH_4_ (mL)	53.0 ^b^	50.8 ^ab^	51.4 ^b^	47.7 ^a^	1.12	0.013	0.527
CH_4_/VFA (mL/mol)	7.89	8.07	8.11	7.99	0.158	0.642	0.376
AFOM (mg/g) ^3^	593 ^b^	557 ^ab^	559 ^ab^	523 ^a^	12.3	0.004	0.965

^a,b,c^ Within each row, the means with different superscripts are different (*p* < 0.05). ^1^ PGP: potential gas production; *c*: fractional rate of gas production; *Lag*: time needed to start gas production; AGPR: average gas production rate; DMED: dry matter effective degradability; ^2^ SEM: standard error of the mean; ^3^ apparently fermented organic matter was calculated from VFA production according to Demeyer [28].

**Table 7:** Gas production and fermentation parameters of diets for medium-producing dairy sheep without almond hulls (MP0) and with almond hulls at 8 (MP8), 16 (MP16), and 24% (MP24) of the fresh matter at 9 and 24 h of in vitro incubation with ruminal fluid from sheep.

Item	MP0	MP8	MP16	MP24	SEM ^2^	*p*=	
						Lineal	Quadratic
Gas production parameters ^1^							
PGP (mL/g dry matter (DM))	259	257	257	264	4.9	0.664	0.447
*Lag* (h)	0.058 ^b^	0.058 ^b^	0.057 ^b^	0.052 ^a^	0.001	0.008	0.060
*c* (%/h)	1.75 ^b^	1.69 ^b^	1.66 ^b^	1.09 ^a^	0.10	<0.001	0.028
AGPR (mL/h)	9.49	9.40	9.33	9.16	0.21	0.294	0.851
DMED (%)	34.6 ^ab^	35.2 ^b^	34.9 ^b^	33.6 ^a^	0.36	0.066	0.030
Fermentation parameters (9 h)							
Total volatile fatty acids (VFA; mmol/g DM)	3.93	3.91	3.70	3.87	0.089	0.379	0.316
Molar proportion of VFA (mol/100 mol)							
Acetate (Ac)	65.7 ^c^	65.2 ^bc^	64.3 ^b^	62.4 ^a^	0.36	<0.001	0.078
Propionate (Pr)	21.4 ^a^	22.2 ^ab^	22.8 ^b^	24.5 ^c^	0.39	<0.001	0.298
Butyrate	9.34	9.21	9.56	9.79	0.181	0.067	0.344
Isobutyrate	0.658	0.370	0.675	0.668	0.1276	0.572	0.301
Isovalerate	1.75	1.63	1.63	1.67	0.064	0.469	0.240
Valerate	1.18 ^c^	1.36 ^b^	1.04 ^a^	0.96 ^a^	0.028	<0.001	0.002
Ac/Pr (mol/mol)	3.08 ^c^	2.94 ^bc^	2.83 ^b^	2.57 ^a^	0.015	<0.001	0.285
NH_3_-N (mg/L)	143	138	137	142	3.8	0.814	0.183
CH_4_ (mL)	16.9	16.8	16.9	17.1	0.43	0.726	0.656
CH_4_/VFA (mL/mol)	4.37	4.33	4.59	4.43	0.430	0.586	0.742
AFOM (mg/g) ^3^	336	335	318	334	0.3	0.472	0.279
Fermentation parameters (24 h)							
pH	6.87 ^a^	6.86 ^a^	6.89 ^b^	6.90 ^b^	0.005	<0.001	0.244
Total VFA (mmol/g DM)	6.69 ^c^	6.40 ^b^	6.09 ^a^	6.11 ^a^	0.078	<0.001	0.078
Molar proportion of VFA (mol/100 mol)							
Acetate (Ac)	64.6 ^c^	64.1 ^bc^	63.3 ^ab^	62.6 ^a^	0.39	0.003	0.863
Propionate (Pr)	19.2 ^a^	20.2 ^b^	20.6 ^b^	21.5 ^c^	0.18	<0.001	0.843
Butyrate	11.9	11.5	12.3	12.1	0.25	0.311	0.636
Isobutyrate	1.20	1.06	1.08	1.07	0.095	0.395	0.496
Isovalerate	1.78	1.65	1.67	1.65	0.041	0.076	0.204
Valerate	1.26 ^b^	1.54 ^c^	1.12 ^a^	1.12 ^a^	0.024	<0.001	<0.001
Ac/Pr (mol/mol)	3.36 ^c^	3.18 ^b^	3.07 ^ab^	2.91 ^a^	0.049	<0.001	0.786
NH_3_-N (mg/L)	223 ^b^	188 ^a^	183 ^a^	191 ^a^	3.6	<0.001	<0.001
CH_4_ (mL)	50.2	46.7	46.1	47.2	1.55	0.204	0.176
CH_4_/VFA (mL/mol)	7.50	7.28	7.57	7.76	0.315	0.471	0.530
AFOM (mg/g) ^3^	583 ^c^	556 ^b^	531 ^a^	536 ^a^	6.3	<0.001	0.033

^a,b,c^ Within each row, the means with different superscripts are different (*p* < 0.05). ^1^ PGP: potential gas production; *c*: fractional rate of gas production; *Lag*: time before starting gas production; AGPR: average gas production rate; DMED: dry matter effective degradability; ^2^ SEM: standard error of the mean; ^3^ apparently fermented organic matter was calculated from VFA production according to Demeyer [28].

## Data Availability

Data are contained within the article.
